# Enhancing children's motivation and enjoyment in playing games: the impact of multi-set game structures in handball-based activities

**DOI:** 10.3389/fspor.2025.1711508

**Published:** 2025-11-28

**Authors:** Frowin Fasold, Fabian Lukac, Stefanie Klatt

**Affiliations:** Section Cognition in Team Sports, German Sport University Cologne, Cologne, Germany

**Keywords:** sport games, school, participation, need for competences, team handball

## Abstract

**Introduction:**

In training sessions or physical education units, the outcome of competitive games—winning or losing—directly affects the enjoyment of the game and children's perception of competence. Sports games are typically structured as single-set games with fixed playing times and cumulative results (e.g., handball, basketball) or as multi-set games with independently scored sets (e.g., volleyball, tennis). Multi-set structures allow for the possibility that winning individual sets, even in an overall defeat, can positively influence perceived skills and enjoyment. This exploratory study investigates whether changing the structure of a handball game (single-set into multi-set) influences children's enjoyment and motivation to play game again.

**Method:**

A 2 × 2 design was employed to evaluate the effects of game structure (single-set vs. multi-set) and match outcome (win vs. loss) on enjoyment and motivation. The participating children (*N* = 122, 50 male, 72 female; age *M* = 10.91 years, *SD* = 0.72) played a handball based small-sided game four times: twice in a single-set structure (10-minute playing time) and twice in a three-set structure (2 × 4 min + 1 × 2 min). After each game, participants rated their enjoyment, motivation to play again, and whether they had won or lost on Likert scales.

**Results:**

While the effects of winning and losing were similar in both game structures, playing in a three-set structure had a positive impact on overall enjoyment and more participants felt motivated to play the game again.

**Discussion:**

The results suggest a positive effect of playing in multi-set structures. Thus, we recommend structure modifications for conducting small-sided games in team sports like handball in children's competitions, training or physical education settings. However, further research on changes in playing structures is warranted.

## 1 Introduction

Regular physical activity, independent of organized or non-organized settings, is crucial for children's overall health and well-being, as it promotes both physical and mental health (1). In organized settings physical education in school (PE) plays a vital role in providing children with opportunities to engage in regular physical activity like memberships in sports clubs. PE offers a structured environment for physical engagement and has the potential to address the psychological factors influencing participation (2). Student motivation is a critical determinant of their engagement in PE (3). In accordance with the Self-determination Theory [SDT, (4)], students' motivation in PE increase when they experience enjoyment and their basic psychological need for competence is met (5). The satisfaction of the need for competence is essential for motivating children to participate in PE with enthusiasm and perseverance (6). A natural way to experience competence in PE is through winning games (e.g., handball based small-sided games), which fosters an intrinsic drive to engage in physical activities both at school and during leisure time (7). This intrinsic motivation is a significant factor in maintaining an active and sport connected lifestyle (8).

Game-based approaches in PE, or in general team sport environments, emphasize the promotion of intrinsic motivation, based on the principle that the most significant learning experiences occur within the game itself [e.g., (9)]. One method for achieving both competence and enjoyment in sports is through small-sided games. These are modified versions of official games played with fewer players on each team and often in smaller playing areas (10). Small-sided games offer several benefits for young athletes, including enhanced skill development and increased enjoyment of the sport. They are often more enjoyable because players are more involved in the action and have more opportunities to succeed (11). With fewer players, a smaller playing area and age/ability adequate rules, individuals have more chances to engage with the ball, make decisions, and play a significant role in the game, positively impacting student motivation (12). Small-sided games can be easily adjusted to suit the age, skill level, and goals of the players, making them a valuable tool in PE [e.g., (9)] or in training settings in sports clubs.

Adjustments and scaling's of the play and game environment for children have shown to favorably impact their motivation and enjoyment. However, research so far has been limited to modifications in field/court area, field/court length, player density (individual field/court area per player), ball size, goal size, and match duration (13). Another possible way to adjust the game might be to change its structure (14). The conventional structure of many team sports, such as soccer and team handball, involves playing for a fixed period and determining the winner by the final score (one-set structure). However, other sports, such as volleyball or beach handball, use a structure where the winner is determined by the number of sets won (multi-set structure). The impact of changing the game structure from a one-set to a multi-set format on player enjoyment and motivation has not yet been investigated.

The possible effects of the adjustment of games structures (one-set vs. multi-set) on the psychological variables of children (enjoyment and motivation) can be explained in accordance with theoretical frameworks and models from social psychology and movement science.

The Self-determination theory (SDT), proposed by Deci and Ryan (15), asserts that individuals possess three inherent psychological needs: autonomy, relatedness, and competence. Due to the specific topic and assumptions of our study, the following description focuses solely on the needs for autonomy and competences, while still acknowledging the relevance of relatedness within the framework of SDT. Small-sided games, such as handball-based activities, show a high potential to satisfy children's need for relatedness, since they are played in teams and involve many cooperative actions. When conducting such activities, the other two needs are more substantial from the perspective of the SDT. Autonomy reflects the desire to experience a sense of freedom and control over one's actions. When individuals perceive themselves as autonomous in their decision making, they are significantly more likely to be intrinsically motivated, undertake activities with enthusiasm, and feel a sense of responsibility and ownership towards those activities (4). The need for competence involves the aspiration to feel efficient and capable in one's pursuits. When individuals perceive themselves as proficient in a particular domain, they are more inclined to engage actively, confront challenges, and persist despite obstacles. In the context of sport activities, the satisfaction of the need for competence is particularly crucial. According to Bureau et al. (16), the need for competence has the strongest association with students' autonomous motivation in PE. Therefore, it is essential for PE teachers or coaches in club settings to create an environment where children can experience success and recognize their learning achievements. A clear indicator of success could be winning a game, while losing may frustrate the need for competence and potentially undermine autonomous motivation.

Promoting self-determination positively affects not only motivation but also the motor learning process. The connection between motivational processes and successful skill acquisition or motor learning is outlined by an additional theoretical framework: The OPTIMAL-Theory, presented by Wulf and Lewthwaite (17), explains motor learning and performance processes in sports. Complementing SDT, this theory highlights the significance of cultivating intrinsic motivation in learners, proposing that learners excel when they are intrinsically motivated, engaging in activities simply because they find them pleasant and captivating. This aligns with SDT's emphasis on autonomy and competence as factors promoting intrinsic motivation. The OPTIMAL-Theory also emphasizes the role of attentional focus in motor skill acquisition. It posits that an external focus of attention (concentrating on the outcomes or effects of movements) is more efficient than an internal focus (concentrating on body movements) in enhancing skill learning and performance (17). This notion aligns with SDT's concept of autonomy, as students feel more empowered when they can focus their attention on external task-related cues.

Both SDT and OPTIMAL-Theory highlight the importance of motivational aspects in sportive behavior and motor learning. Consequently, learning environments in game based activities, independent if conducted in PE or club settings, should be structured and organized in ways that fulfil childrens' need for competence, their autonomy and facilitate an external focus of attention. To design such learning environments, the Constraints-Led-Approach (CLA) represents a useful tool. The CLA embodies a coaching and teaching method frequently employed in sports (18–20). The CLA considers three types of constraints: individual constraints (athletes' physical and psychological characteristics), environmental constraints (external factors like weather, equipment, and playing surface), and task constraints (specific aspects of the sport, such as rules, goals, and opponent behaviors). Existing literature already imply the CLA, manipulating these constraints to make sports more age-appropriate and attractive for children. For instance, Dieu et al. (19) implemented various modifications to task and environmental constraints in the organizational planning of badminton games, aiming to enhance students’ physical activity and enjoyment. Fasold et al. (14) and Engel et al. (21) demonstrated the necessity of modifying (i.e., scaling) children's sports, and Broadbent et al. (13) extensively discuss the impact of such changes on motor performance. However, it remains unclear how the above mentioned adjustments of game structures, which represents a simple task constraint in the context of CLA [e.g., (18)], could potentially enhance motivation—a crucial aspect of motor learning (17).

Summarizing the theoretical background, with a simple task modification by changing games from one-set competitions to multi-set competitions (following the CLA), the specific needs of children could be satisfied. If game-based activities are played in a competitive setting, in the one-set structure, there are winners and losers at the end of the playing time. This affects children's needs for competence and enjoyment either positively or negatively. In the multi-set structure, while there are also winners and losers at the end, the losers may have an increased possibility of experiencing a sense of success along the way (e.g., by winning a set despite losing the game overall). According to the theoretical assumptions [SDT, (4)], this increase possibility of meeting their need of competence could enhance their physical activity enjoyment (5). These task adjustments could also help direct the students' attentional focus externally. It is anticipated that playing in multiple shorter sets could help focus more on task-related cues (e.g., the result, deciding the set), which is associated with increased successful motor learning [OPTIMAL-theory, (17)] and an increase in intrinsic motivation and enjoyment. These increased satisfactions of the needs of children increasing the probability of ongoing participation in sports activities [e.g., (7)], and as factors in fostering an active lifestyle (8).

Thus, the aim of our explorative study is to evaluate, for the first time, the effects of an adjusted game structure (multi-set structure) of a handball-based small-sided game on students' perceived enjoyment and motivation.

## 2 Method

### 2.1 Participants

Due to the fact that no prior research on this topic exist, no *a priori* power analysis was conducted to determine the sample size. Thus, the sample size was estimated and determined by the number of participating groups of children (school classes). The institutional ethics commission previously approved this approach.

The study was conducted as a regular part of PE lessons for four classes (5th and 6th grades) from three different German secondary schools. A sample of 214 students participated in these lessons. Informed consent was obtained from all participants prior to the study, and parental consent was present for 122 students (50 boys: 41%, and 72 girls: 59%). The average age of the final sample (*N* = 122) was 10.91 years (SD = 0.72).

Ethical approval was granted by the ethics committee of the German Sport University Cologne (043/2022) before any data collection took place. Students voluntarily participated in the study. Participants completed a paper-and-pencil survey after each small-sided game they played.

### 2.2 Design

In a 2 × 2 design, the effects of the within-subject factors *game structure* (one-set vs. three-set structure) and *match outcome* (win vs. loss) on the dependent variables *fun* (enjoyment) and *motivation* (to practice the game again) were evaluated. To simplify the evaluation of the research topic with participants in this age group, and due to the exploratory nature of the investigation, two simple measurement levels were used (metric for fun, categorical for motivation) and where evaluated independently.

The authors acknowledged that evaluating the two variables with different measurement scales does not permit a dependent statistical analysis, but prioritized the simplification for the study's conduct over strict statistical requirements. Furthermore, predicting the number of participants in each experimental condition was not possible prior to the study due to the random outcomes of the games. Therefore, the observable effects in the descriptive data are considered more important than the *post-hoc* calculated effect sizes and statistical significance.

### 2.3 Procedure

The study was conducted over two consecutive PE lessons in the four participating classes. It was found that PE classes are not suitable for the implementation of the regular game of handball in a fixed structure, so the level of difficulty was reduced by using the small-sided game of mat ball. The rules, equipment (ball, size 1) and objective of the sport remained unchanged, thereby creating a handball-like variant suitable for the study. Mat ball is a small-sided game played between two teams of five players each. The participants were randomly assigned to their teams at the beginning of the first lesson of one class (participants were assigned to the teams by drawing numbers), and the team composition remained stable over the two PE lessons. Following a tournament system, each team competed against every other team during a PE lesson. Depending on the number of students in each lesson, four or five teams participated in the tournament.

The court measures 20 × 16 m, and the duration of each game was ten minutes. A jump ball in the center of the court initiates the beginning of each match. The aim of the game is to place the ball onto the soft floor mat (foam landing mat) of the opposing team, using the rules of handball. The mats are positioned two meters from the baseline, enabling players to approach them from all angles. Every placement of the ball on the mat earns a single point. A player can retrieve the ball while on the defensive during its airtime, such as bouncing or passing. The rules prohibits any physical contact, the violation of which results in a change of possession.

The matches were conducted in both a one-set structure (the team with the higher score after ten minutes won the game) and a three-set structure (two sets of 4 min and one set of 2 min). In the three-set structure, the sets are counted independently, and the possible results are 3:0 ore 2:1. No match could end in a tie. If the game time was over and the score was a tied, a golden goal situation was played to decide the match or the set. The order of the game structures was randomly determined at the beginning of both PE lessons.

After every match, participants fulfilled a simple questionnaire by responding two scales. The perceived level of fun was assessed by the question “How much fun did you have playing the game?”, using a five-point Likert smiley-scale, ranging from a very happy looking face (1) to a very sad looking face (5). The motivation was assessed by the question, “Would you want to practice the game again?” The available answers were “Yes”, “No” and “I am not sure”. In a third question, the participants responded if the match was a win or a loss.

### 2.4 Statistical analysis

To assess the impact of the within-subject factors on the dependent variables, we conducted an initial descriptive analysis. This involved calculating the means (including standard deviations) of the ratings of all participants for the variable *fun*, and determining the frequency distribution of the variable *motivation* across the four experimental conditions (one-set/win; one-set/loss; three-set/win; three-set/loss).

The descriptive data for the dependent variable *fun* were verified using *post-hoc* ANOVA with repeated measurements, incorporating data only from participants who satisfied all four experimental conditions. If more than one response was present for a participant in a given condition, a mean value was calculated. For displaying the effect size, *η*^2^_partial_ was calculated.

In terms of the variable measuring *motivation*, the frequency distribution of responses (all responses) across the various experimental conditions was compared through a simple test of equal distribution (*χ*^2^-test). For reporting the effect size, Cramers *V* was calculated.

## 3 Results

Due to the study design, the number of results in the experimental conditions was unpredictable prior to conducting the study. Consequently, the statistical analysis was conducted in two steps: descriptive presentation and inferential analysis. The results for the factors *fun* and *motivation* (to practice the game again) were presented separately.

### 3.1 Fun

Table 1 presents the mean responses of all participants across each experimental condition. The study results reveal that winning is more enjoyable than losing in both game structure. Furthermore, the data indicate that in a three-set structure, both winning and losing are perceived as slightly more enjoyable compared to a one-set structure.

**Table 1:** Descriptive presentation of means and SDs of all participants in the different experimental conditions for the dependent variable fun.

		Game Structure	
		*Fun* in the One-Set Structure	*Fun* in the Three-Set Structure
Match Outcome	Win	1.86 (0.99) *n* = 75	1.52 (0.73) *n* = 75
	Loss	3.07 (1.27) *n* = 79	2.72 (1.14) *n* = 75

The values represents the ratings on the five-point Likert-smiley-scale (values: 1 = very happy,…, 5 = very sad).

As reasoned in the statistical analysis section, the inferential analysis was focused on these participants (*n* = 17) who provided results in all four experimental conditions (Table 2). A MANOVA was conducted, which showed no significant effect of the interaction of the factors *match outcome* and *game* structure, on the dependent variable, *F*(1, 16) = 0.76, *p* = .39, *η*^2^_partial_ = .04.

**Table 2:** Mean and SD for the dependent variable fun of the reduced sample (*n* = 17) participating in all of the different experimental conditions.

		Game Structure	
		*Fun* in the One-Set Structure	*Fun* in the Three-Set Structure
Match Outcome	Win	1.82 (1.05)	1.23 (0.43)
	Loss	3.08 (1.25)	2.85 (1.01)

The values represents the ratings on the five-point Likert-smiley-scale (values: 1 = very happy,…, 5 = very sad).

A main effect of the factor *match outcome* was found, *F*(1, 16) = 36.88, *p* < .001, *η*^2^_partial_ = .69 (win *M* = 1.52, *SD* = 0.75; loss *M* = 2.97, *SD* = 1.13), and a main effect of the factor *game structure* was observed, *F*(1, 16) = 3.35, *p_one−tailed_* = .04, *η*^2^_partial_ = .17 (one-set structure: *M* = 2.45, *SD* = 1.15; three-set structure: *M* = 2.04, *SD* = 0.72). The interpretation of the descriptive results of the entire sample (Table 1) and analysis of variances of the reduced sample (Table 2) yield the following findings: Firstly, winning is more enjoyable than losing, regardless of whether the game is played in a one-set or a three-set structure. Secondly, playing games in a three-set structure is generally more enjoyable, but losing is equally less enjoyable as in the one-set structure.

### 3.2 Motivation

Regardless of the experimental condition, responses to the question “Would you want to practice the game again?” (*n* = 481) showing the following unequal distribution [*χ*^2^(6, 481) = 32.49, *p* < .001, *V* = .18]: “Yes” = 52.60%, “No” = 21.40%, “I am not sure” = 26.00%,.

Winning and losing have a comparable impact in both conditions and the choose of the responses of the participants [one-set structure, *χ*^2^(2, 254) = 17.22, *p* < .001, *V* = .26; three-set structure, *χ*^2^(2, 227) = 9.86, *p* = .007, *V* = .20]. The category “Yes” was chosen more frequently when participants won the game (Table 3).

**Table 3:** Frequency distribution of the response categories by answering “Would you want to practice the game again?” in the different experimental conditions.

Response Category	Game Structure			
	Practice again? One-Set Structure		Practice again? Three-Set Structure	
	Win (*n* = 127)	Loss (*n* = 127)	Win (*n* = 118)	Loss (*n* = 109)
Yes	60.6%	34.6%	64.4%	51.4%
No	19.7%	31.5%	9.3%	24.8%
I am not sure	19.7%	33.9%	26.3%	23.9%

Comparing the effect of winning in the one-set and three-set structure (Table 3), slight differences in the distribution of responses are visible, but these have no significant effect, *χ*^2^(2, 245) = 5.77, *p* = .06, *V* = .15.

When comparing the effect of losing in both game structures, the visible differences (Table 3) in the distribution of responses result in a significant effect, *χ*^2^(2, 237) = 6.81, *p* = .03, *V* = .17. If participants lose in the three-set structure, over half of the responses are “Yes”, whereas losing in the one-set structure leads to nearly an equal distribution of responses across all categories.

## 4 Discussion

The aim of this study was to evaluate whether playing in the three-set structure has a positive effect on students’ perceived fun and the motivation to play again in a small sided game compared to playing in a one-set structure. Winning and losing have a quite similar effect on the perceived fun in the game in both structures, but there is a slight indication that playing in the three-set structure is associated with more enjoyment. Regarding the motivation to play again, the three-set structure seems to be supportive on ongoing participation. If participants lose in the three-set structure, more than the half of responses are “Yes” to play again, whereas losing in the one-set structure leads to decline in the “Yes” responses and show nearly an equal distribution of responses across the categories. This could be attributed to the fact that the losers in the three-set structure have more hope of achieving success (winning a set or match) in a future match under the same condition.

Whereas the effects are small and the results are based only on simple quantitative measures, the study shows that a simple modification in the task could influence psychological factors of the participants. Nevertheless, these effects could be explained based in ideas from theories from different learning and teaching associated research areas, such as social psychology or movement science [e.g., SDT (4); OPTIMAL-Theory, (17)]. These effects are particularly interesting for all kind of sports activity practice, because previous studies have suggested that enjoyment in PE is a missing link between motivation in school PE and ongoing participation in sports [e.g., (22)]. Thus, even though the effects are small, it seems plausible to simply modify competitions in handball-based small-sided games (like the presented mat ball) by playing them in a multiple-set-structure rather than in one-set structure.

Following the idea of the CLA [e.g., (20)], modifications of the constraints in a learning process (e.g., a handball game) are an elementary part for the design of effective and efficient learning environments [e.g., (9)]. Modifications of the metrics of a game (e.g., field size, ball equipment), as proposed by the body-scaling approach of Broadbent et al. (13), are well evaluated and, if applied correctly, effective in supporting the learning process and the motivational factors around sport participation. Based on this, we could now show for the first time that a simple modification of the task constrains by adapting the game structure can in the same way positively influence the effects of playing activities.

Nevertheless, some limitations of our study should be noted. First, the effects were measured using simple quantitative tools (Likert scales) that are not validated for the specific research topic. Thus, we must emphasize the exploratory approach of this study, and further investigations are necessary to confirm and extend our results. Ongoing research could explore this topic using age-appropriate qualitative methods such as interviews, structured observations, or mixed-methods designs [e.g., (23)]. Additionally, validated quantitative measures like the Sport Commitment Questionnaire-2 (24) could be used to replicate and validate the results. Furthermore, the effect of winning a set in the three-set structure was not evaluated (the final outcome 3:0 or 2:1 was not recorded). Only whether the result was a win or a loss was recorded, as the sole aim of this study was to focus explicitly on the effect of a win or loss. But, it remains unclear if there are different effects on enjoyment and motivation when participants win a set despite losing the overall game. The same applies to the one-set structure: it is conceivable that a loss or a win with a large score difference (e.g., finals score 10:2) has a different effect than a narrow score at the end of the game (e.g., 10:9). Therefore, to gain deeper insight into the effects of modifying game structures on enjoyment and motivation, future research should additionally consider the final score in relation to the outcome of the competitions. To increase statistical power, future research should increase the sample size in all conditions of the study design. Due to the unpredictable nature of this design, where the outcome and thus the condition are not known until a match is finished, an open series of matches should be planned. Data collection should only finished once all participants have experienced all experimental conditions (one-set vs. three-set structure × win vs. loss). To enhance the probability of balanced wins and losses, the skill levels of the teams should be balanced. A quasi-experimental design could be employed, where participants are selected *a priori* based on their skills to achieve more balanced teams. Furthermore, focusing on sociodemographic variables such as sport-specific experience, age, or sex could strengthen internal validity.

In general, with this study, we did not aim to discuss the value and appropriateness of competitions in PE. There are many arguments for banishing competitive structures from PE, but similarly, there are many arguments that competition can be a valuable aspect of PE. This discussion is ongoing in pedagogy and philosophy [e.g., (25)], and our study does intend to determine whether competitions should be part of PE or not. Our study can only demonstrate that, if competitions are used in PE, simple modifications can make them more suitable for one of the aims of PE: fostering general physical activity [e.g., (2)]. Such modifications or adaptions are a normative argument in the discussions surrounding the competitive nature of sport in school (25).

Next to the discussion about competitions in PE, small-sided games in club training are generally conducted in a competitive manner, as athletes should also be prepared for the psychological demands of league or tournament structures. Changing the format from single-set to multi-set competitions in training increases the likelihood of high-pressure situations (e.g., crunch time in balanced scores shortly before the end of the game/set), thereby providing opportunities to practice psychological skills for handling such scenarios.

### 4.1 Practical implications

Based on the conducted study and the results shown, we suggest the use of multiple-set structures in the design of game competitions in sport activities like PE or training practice in clubs. We particularly recommend these for small-sided games where a high density of events (e.g., goals, scores) is expected, which are typical of sports such as team handball. If the expectation of scoring is quite low and the games end with low scores (e.g., 1:0, 2:1), the probability of a tie is high, and determining winners and losers via a golden goal could take a lot of playing time. Therefore, to provoke the positive effect on fun and motivation through the multi-set structure, the task constraints of the small-sided games must be designed in such a way that multiple scores are possible, simplifying the detection of success within short playing times (e.g., 4 min). A general balancing of the success of offensive (success-rate in scoring) and defensive (success-rate in defending) behavior in the design of small-sided games [proposed by (9)] could strengthen the development of rich learning conditions and, therefore, the development of joyful and motivational learning climates.

## Data Availability

The datasets presented in this study can be found in online repositories. The names of the repository/repositories and accession number(s) can be found below: OSF, doi: 10.17605/OSF.IO/R9DZK.
